# 

**Published:** 1896-11-14

**Authors:** 


THE HOSPITAL. Nov. 14TH, 1896.

ABSOLUTELY PURE, therefore BEST,

CADBURY'S COCOA

"The standard of highest purity at present attainable."—*Lancet*.

CADBURY'S COCOA

NO ALKALIES USED.

### THE HOSPITAL

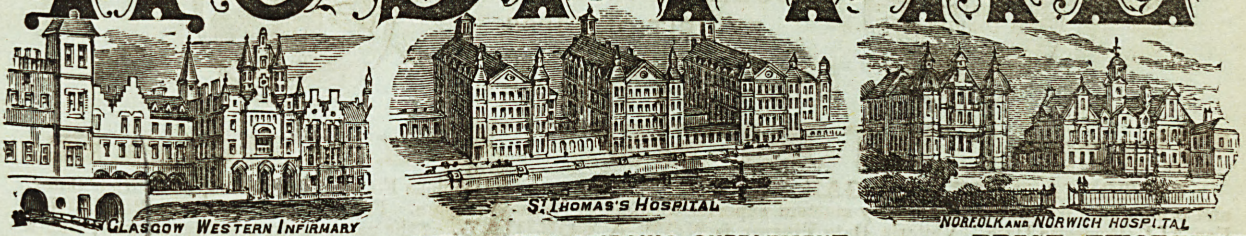

No. 529. Vol. XXI.

SATURDAY, NOV. 14TH, 1896.

WITH EXTRA NURSING SUPPLEMENT.

[Registered at the General Post Office as a Newspaper for transmission in the United Kingdom and Abroad.]

PRICE TWOPENCE.

Per Annum, 10s. 6d., post free.

Monthly Parts, 6d.; post free, 8d.

Ditto per Annum, 8s. 6d., post free.

